# The Profession and Titular Distinctions

**Published:** 1916-01-15

**Authors:** 


### The Hospital

The Workers' Newspaper of Administrative Medicine and Institutional Life, Administration, National Insurance and Health.

No. 1544, Vol. LIX. SATURDAY, JANUARY 15, 1916. PRICE ONE PENNY.

### THE PROFESSION AND TITULAR DISTINCTIONS.

The nomination of individual citizens as persons upon whom may be appropriately conferred various decorations or titles of honour is usually regarded as a proceeding which must involve a very anxious responsibility. Apart from the pressure of candidates or their supporters, and the question Aye or No, there is the delicate adjustment of grade or degree as this is deemed appropriate in each particular instance. The grades and degrees are numerous, and to the outsider it would seem to be a bewildering task to distribute them with a nice accuracy and with strict justice. Possibly there exists machinery, unknown to the uninitiated, by which the task is lightened, and there is at least the comforting consideration that the decisions, whatever they be, have not to be provided with any public defence. Somehow or other the list with its inclusions and omissions is compassed, and whatever private criticisms may be cultivated, open agitation makes no sign. The fortunate ones wear their honours bravely and as to the manner born, and the disappointed wisely conceal their annoyance. For those who are ranged neither in the one class nor in the other there remains the opportunity for congratulation—or it may be for sympathy—and the consciousness that ancient traditions have been maintained and a pleasing variety introduced into social life. In all these incidents, both active and passive, members of the medical profession, like other citizens, have their share, and we imagine that as a class they stand much in the same position as their fellows. The ambitious, the disappointed, the gratified, and the unconcerned are doubtless all represented in the ranks of medicine just as they are represented in the community generally, and, so far as we can judge, there is not in the profession any undue proportion of any one of these various groups. Though everyone may not be satisfied, there is probably as much satisfaction as can reasonably be expected in an admittedly imperfect world.

While offering this expression of general contentment, or at least of resignation, we cannot altogether refrain from expressing a wish that the titular honours which come to the profession could be so arranged as to correspond with a difference which is beyond question both real and important Medical practitioners in their capacity as citizens take their opportunity or chance of such honours on the same platform as their neighbours. But, in addition, a certain small number of them receive recognition, not for what may be called civic or personal reasons but purely on account of professional eminence or achievement. It would certainly be convenient were the titles conferred on these two groups to be such as to indicate the difference which exists between them. This is not to say that either one group or the other does not merit recognition or honour; nor is it to propose that one is more worthy than the other. Still less, if less may be, is it to quarrel with individual instances or with the general distribution of honours to the medical profession. The plea is merely for the establishment of a distinction corresponding to an essential difference. According to the existing custom there is no recognition of this difference. All come under a common designation, and there is as a consequence no identification of the particular kind of merit which has earned and received an authoritative approval. This, we venture to imagine, cannot be altogether satisfactory to the wearers of the honours thus conferred, and it both confuses the public mind and deprives the State of an opportunity of individualising the services rendered to its interest. Without being invidious we may take one or two examples. A medical practitioner, even as any of his fellow-citizens, may become prominent in municipal life and may perhaps for one or more years occupy the mayoral chair, and this at a time when some considerable incident is associated with the borough over which he presides. Out of such a combination of events may come to him, say, the honour of knighthood. No one will grudge him the distinction or will doubt that it has been well earned.

Again, personal and professional relations may exist between a practitioner and some prominent personage, political or otherwise, and in accordance with many precedents the bestowal of some titular distinction may follow. We make no quarrel with the custom, which indeed has many pleasing features. Our point is rather that while such cases as those we have just instanced need not fail, it would be an advantage were the mark applied to them different from the one selected for those whose claim to attention rests solely on professional distinction.

On the face of things it argues some poverty of resource when the State recognition of merit indicates with one and the same label the work of a successful mayor, the wise medical counsel of a private professional adviser, and the achievements, say, of a Jonathan Hutchinson. All alike have their merit, and all may perhaps be appropriately recognised, but the merit is not of one and the same order, and consequently the recognition ought not to be of one and the same order either. There is no need to enter on an analysis for the purpose of putting such varied services in a definite rank or precedence, or with a view to decide which is of the greater value to the community. Let it be allowed that each in its own way is of value and that each merits recognition. But when this is granted it still remains true that there exists an opportunity for differentiation resting, not on a comparison of degree of merit but on an appreciation of the kind of merit. Possibly if we entered on particular forms of reward we might seem to disparage some and to exaggerate others. But our advocacy is limited to the principle that, whatever be the details, it would be well were attainments purely professional and scientific recognised in a different fashion from that extended to more general and extra-professional triumphs.

